# Maternal belief in a just world and moral commentary during picture book reading in infancy

**DOI:** 10.3389/fpsyg.2026.1774557

**Published:** 2026-05-04

**Authors:** Takeshi Kishimoto, Asuka Kurihara

**Affiliations:** Department of Psychology, Faculty of Liberal Arts, University of the Sacred Heart, Shibuya-ku, Tokyo, Japan

**Keywords:** belief in a just world, caregiver-toddler interaction, developmental moral cognition, early moral development, moral socialization infancy, parental moral beliefs, parental worldview transmission, shared picture book reading

## Abstract

**Objective:**

This study examined whether caregivers’ picture book reading styles involving moral narratives are associated with the strength of their belief in a just world.

**Methods:**

We investigated the relationship between caregivers’ just-world belief scores and the number of belief-related utterances produced beyond the picture book text. We observed picture book reading sessions involving 24 infants or toddlers and their mothers, extracted maternal off-text commentaries reflecting just-world beliefs, and examined their association with mothers’ belief strength.

**Results:**

The number of maternal narrative additions related to belief in a just world was significantly and positively correlated with belief in ultimate justice.

**Conclusion:**

The finding suggest that mothers with stronger belief in ultimate justice tend to emphasize just-world themes during picture book reading.

## 1 Introduction

People tend to think that unfortunate events result from past misdeeds. People also sometimes believe that present hardships will be compensated for by future success. Despite the inability to identify clear causal relationships between past and present events or between present and future events, people often assume that such causality exists. This assumption stems from the belief that people receive what befits them. The belief that the world is a fair and safe place, where individuals are not suddenly struck by misfortune and receive outcomes they deserve, is referred to as the belief in a just world (Lerner, 1980; Sun et al., 2024). Two forms of just-world belief have been identified: belief in immanent justice and belief in ultimate justice. Belief in immanent justice refers to the tendency to attribute current misfortune to past wrongdoing. By contrast, belief in ultimate justice refers to the tendency to believe that losses caused by injustice will eventually be compensated by future good fortune (Hafer and Bègue, 2005).

Based on this belief, humans interpret positive outcomes, such as goodness, happiness, and success, and negative outcomes, such as evil, misfortune, and failure, within a unified framework and attempt to construct causal relationships within it (Lerner, 1980). For example, people tend to believe that doing good deeds will bring good things back to them—such as “If you turn in a lost item to the police box, something good will happen to you someday”— and that engaging in wrongdoing will bring bad things upon themselves—such as “If you steal money from a found wallet, you’ll be punished someday.” These beliefs allow individuals to live without constant anxiety about unpredictable or unreasonable misfortune, such as suddenly dying the next day. They also support sustained investment in effort, training, and study by reducing fears that such efforts will be futile (Lerner, 1980; Hafer and Bègue, 2005). Conversely, belief in a just world can also give rise to victim blaming. In the world implied by this belief, “misfortune should befall those who have done wrong.” When innocent individuals suffer harm, this threatens the belief in a just world. To preserve this belief, people may blame innocent victims by assuming that they must have been responsible for their misfortune (Hafer, 2000; Kaplan, 2012).

From an evolutionary psychological perspective, belief in a just world has been conceptualized as a psychological mechanism underlying reciprocal altruism in humans, defined as the exchange of altruistic behavior with others (Igou et al., 2021). In a just world, good deeds that temporarily disadvantage the self for the benefit of others are expected to be compensated later. Individuals who strongly endorse just-world beliefs may therefore view helping behavior as an investment that will eventually yield personal benefit. Classical experimental studies demonstrated that individuals with strong just-world beliefs were more likely to engage in helping behavior toward others in need (Zuckerman, 1975; Miller, 1977). More recently, Igou et al. (2021) revealed that stronger just-world beliefs were associated with intentions to help others through positive emotions and a sense of meaning in life. Together, these findings suggest that belief in a just world, as a cognitive style, may play a significant role in maintaining reciprocal altruism, a key mechanism supporting human social organization.

As outlined above, belief in a just world has been associated with behaviors characteristic of human society, including effort, helping behavior, and victim blaming. By contrast, research on the developmental processes through which this belief emerges remains limited (Tatsi and Panagiotopoulou, 2023; Bartholomaeus and Strelan, 2019). Most developmental studies have focused on adolescents, such as junior high or high school students. For example, Dalbert and Stoeber (2006) conducted an approximately eight-month longitudinal questionnaire survey with German youth aged 14–19, demonstrating that experiences of fair treatment in life domains such as school and home strengthened belief in a just world. Although several other studies have examined just-world beliefs in adolescents, most focused on associations with related variables, including academic performance (Peter et al., 2012), school distress (Dalbert and Stoeber, 2005), and self-esteem at school (Donat et al., 2016). Few studies, aside from Dalbert and Stoeber (2006), directly examined the developmental trajectory of belief in a just world during adolescence (Bartholomaeus and Strelan, 2019). Furthermore, despite the recognized importance of investigating the origins of just-world beliefs in infancy and early childhood, research on these earlier developmental stages remains extremely scarce (Tatsi and Panagiotopoulou, 2023; Bartholomaeus and Strelan, 2019).

Recently, Kanakogi et al. (2025) conducted an experimental cross-sectional study examining the development of belief in a just world in children aged 5–9 years and in adults. Participants were presented with a protagonist who had experienced either a fortunate or an unfortunate event. To assess immanent justice beliefs, participants were asked whether the protagonist had done something good (e.g., helping with cleaning) or something bad (e.g., hitting a friend) the previous day. To assess ultimate justice beliefs, participants were asked whether the protagonist would experience a positive event (e.g., finding a beautiful flower) or a negative event (e.g., being barked at by a dog) the following day. The results showed that even five-year-olds tended to assume that a fortunate protagonist had done something good the previous day, whereas children aged seven years and older increasingly assumed that an unfortunate protagonist had done something bad the previous day, suggesting a complex developmental trajectory for immanent justice beliefs. Conversely, the tendency to expect positive future outcomes for both fortunate and unfortunate protagonists was observed consistently across all age groups, including five-year-olds. These findings suggest that belief in ultimate justice may already be present by approximately five years of age.

The study by Kanakogi et al. (2025) represents one of the few empirical investigations into the early development of belief in a just world and is therefore of substantial importance. However, as the authors noted, there is considerable individual variability in just-world beliefs among adults (Hafer and Correy, 1999), and the developmental origins of this variability remain unclear.

How do such individual differences in emerge? One possibility is that parents’ just-world beliefs are culturally transmitted to their children through socialization processes. Evidence supporting this possibility comes from cross-cultural research. Previous studies showed that Japanese individuals endorsed stronger beliefs in immanent justice than Americans, whereas Americans endorsed stronger beliefs in ultimate justice than Japanese individuals (Murayama et al., 2022). In addition, Murakami et al. (2022) reported cultural differences in belief in just deserts (BJD), a concept closely related to belief in a just world. The strength of this belief was higher in Japan than in the United States, the United Kingdom, China, and Italy.

These findings suggest that belief in a just world may be socially transmitted across generations within communities. Given that the family represents the earliest and most immediate developmental context, it is plausible that parents’ just-world beliefs are transmitted to their children through everyday socialization processes. Empirical support for this hypothesis comes from a survey examining belief in a just world among fathers, mothers, and children within families (Schönpflug and Bilz, 2004). Analysis revealed that children’s just-world beliefs were positively correlated with those of both fathers and mothers. Moreover, children who reported better relationships with their parents, especially with fathers, exhibited greater similarity in just-world beliefs to their parents. These findings suggest that close parent–child relationships and frequent communication may facilitate the intergenerational transmission of just-world beliefs.

The positive correlation between parents and children’s degrees of belief in a just world identified by Schönpflug and Bilz (2004) suggests that this may be transmitted from parents to children through interactions within the family. However, this finding alone is insufficient to demonstrate intergenerational transmission. Correlational evidence does not establish causation. Ideally, causal relationships should be examined through long-term longitudinal studies, and some research has addressed this issue. However, when considering the cultural transmission of beliefs, a micro-level perspective is also necessary alongside this macro-level approach (Kashima, 2016). Specifically, attention should be paid to interactions between infants or toddlers and caregivers and to caregivers’ belief in a just world. It is assumed that the cumulative effects of these micro-level interactions ultimately shape the degree of this belief in a child’s as a developmental outcome. In the transmission of beliefs between parents and children, a process of mutual understanding has been proposed, in which parents actively engage with their children to foster the sharing of common beliefs (Kashima, 2014; Echterhoff et al., 2010). In fact, previous research suggests that parents’ everyday conversations with their children help shape the children’s cultural identity by conveying the forms of discourse valued within that culture (Minami and McCabe, 1995). As members of the society into which they are born, parents are expected to educate their children in every aspect of their daily interactions so that the children can function smoothly and participate actively in the community (Callanan and Siegel, 2014). The stronger a caregiver’s belief in a just world, the more likely they are to view behavior consistent with that belief as desirable within their society. Consequently, such caregivers are more likely to educate their children to behave in ways consistent with a just world belief during their daily interactions.

Research indicates that shared attention with caregivers during activities such as watching movies or reading picture books facilitates moral understanding in infants and toddlers. Shimizu et al. (2018) found that infants aged 15–18 months whose mothers frequently discussed moral evaluations of characters while watching videos featuring “good guys” and “bad guys”—as used in Hamlin and Wynn’s (2011) intuitive moral judgment experiment—exhibited a preference for the prosocial “good guy.” Furthermore, Chen et al. (2025) showed that four-year-olds in an intervention group who read picture books with parents on social themes such as friendship and sharing showed significant improvements in empathy and prosocial behavior, as assessed through questionnaires and experimental tasks, compared with four-year-olds in a control group who read picture books on different themes, such as science and creativity. These findings suggest that young children may deepen their moral understanding based on caregivers’ evaluations of the moral content presented in picture books and videos. Given that belief in a just world serves as a crucial psychological foundation for morality (Igou et al., 2021), children’s acquisition of this belief may also be promoted through media-mediated interactions with caregivers. However, if caregivers’ just-world beliefs are transmitted to children through such interactions, individual differences in caregivers’ beliefs are likely to influence how they engage with moral media. To our knowledge, no previous research has demonstrated that individual differences in caregivers’ belief in a just world affect communication with infants and toddlers through morally themed media.

In this study, we examined whether the degree of a mother’s belief in a just world was related to how she read picture books with themes of rewarding good and punishing evil to her child. Previous research has shown that when caregivers watched videos with moral themes alongside children, they tended to comment on social judgments of good and evil (e.g., Shimizu et al., 2018). Accordingly, this study focused on mothers’ utterances during picture book reading, particularly those that extended beyond the text written in the book. The focus on mothers’ comments beyond the text of picture books stems from the fact that previous research has confirmed that mothers make such comments to help their children understand the content of the books (Muhinyi et al., 2020). We examined whether mothers with stronger just-world beliefs were more likely to add their own evaluations of characters’ morality or to reference a just-world worldview, such as “good triumphs over evil” during narration.

## 2 Methods

### 2.1 Participants

The participants were 24 infants (11 boys and 13 girls) and their mothers. The infants’ mean age was 993 days (minimum: 300 days; maximum: 1683 days; standard deviation: 375.71), and all were at least 9 months old, an age at which joint attention with caregivers becomes possible (Tomasello, 1999). The mothers’ mean age was 35 years (minimum: 28 years; maximum: 45 years; standard deviation: 4.48), and all but one—who did not respond—had a college degree or higher. Two additional mother–child pairs participated; however, one pair was excluded because the toddler refused to participate, and the other pair was excluded because the questionnaire collecting the toddler’s age and other information was not returned. All participants were users of childcare support rooms located in Tokyo, that are available to preschool-aged children and their caregivers. This study was conducted after receiving ethical review and approval from the author’s university (Approval Number: 2023_31).

### 2.2 Materials

The picture book used in this study was *“Anpanman and the Baikinman”* (Yanase, 1979). “Anpanman” is one of the most well-known protagonists created by Takashi Yanase. Anpanman is a hero whose face is made of sweet red bean bun and who fights his arch-enemy, Baikinman, a germ character, to protect the world. The book follows a moralistic narrative structure centered on rewarding good and punishing evil, and every page features illustrations that correspond to the text. The plot was as follows:

*One day, Baikinman covered the sky with dark clouds, sapping people’s energy. Anpanman flew into the sky to clear the clouds, but was defeated by Baikinman. So, Jam Uncle, who created Anpanman, reshaped Anpanman’s face into a new, giant anpan. Transformed into Giant Anpanman, he defeated Baikinman, and the sky cleared up.*

After completing the experimental observation described in the next section, mothers completed a questionnaire assessing belief in a just world. We used the Japanese version of the “Just-World Belief Scale,” originally developed by Maes and Schmitt (1999) and revised by Murayama and Miura (2015) and Umetani et al. (2020), to measure ultimate just-world beliefs (e.g., “The day will come when all victims who have suffered will be rewarded,” and three other items) and immanent just-world beliefs (e.g., “Those who plot evil will be brought down by their own schemes,” and three other items). This scale was a 5-point scale, with “Disagree” rated as 1 and “Agree” as 5. In addition, to determine how often participants read picture books at home, we asked them to specify the number of days per week they read picture books, drawing on the study by Okumura and Kobayashi (2022).

### 2.3 Procedure

The experimental observation was conducted in a room at a childcare support center in Tokyo. In addition to the participating mother and child, two or three other mother–child pairs were freely playing in the room. After the picture book was handed to the participating mother, the observation began. Mothers and children were allowed to read the book in any manner they chose, with no restrictions on behavior. After the mother finished reading the text on the final page, the observation concluded. The entire observation period was recorded using a video camera and lasted approximately 5–10 min.

After the observation, mothers were asked to complete a questionnaire regarding the child’s date of birth, their beliefs about a just world, and how many days a week they read picture books. Some mothers completed the questionnaire at home and returned it by mail.

### 2.4 Coding

The recorded footage was coded as follows. From the moment the mother began reading the text on the first page until she finished reading the text on the final page, all maternal commentaries were transcribed verbatim. Utterances that did not appear in the picture book text were extracted from the transcripts. Following Shimizu et al. (2018), we segmented them based on pauses, syntactic boundaries, and prosodic information. Utterances separated by a gap of one second or longer were treated as independent. When two or more syntactic units appeared within a single utterance without a one-second gap, each unit was counted as independent.

Among the segmented maternal additions to the picture book text, we coded utterances related to belief in a just world, including statements that evaluated characters’ social behavior, described prosocial or antisocial characteristics, and emphasized situations consistent with just-world beliefs. Statements evaluating social behavior included explicit judgments, such as “What a terrible thing to do,” as well as descriptions emphasizing prosocial or antisocial actions, such as “He's punching the bad guy.” Statements describing prosocial or antisocial characteristics referred to evaluations of characters without reference to specific actions, such as “Anpanman is so cool.” Statements emphasizing situations assumed by belief in a just world referred to utterances highlighting the rewarding of good and punishment of evil, such as “Baikinman got defeated,” or emphasizing eventual triumph during scenes of temporary misfortune, such as “Oh no, Anpanman is having a rough time.”

Each segmented maternal commentary was coded related or unrelated to belief in a just world. To assess inter-rater reliability, two coders independently coded the utterances of six mothers randomly selected from the sample of 24, yielding substantial agreement (Cohen’s *κ* = 0.77; Landis and Koch, 1977).

## 3 Results

### 3.1 Reliability of the just-world beliefs scale

First, mothers’ responses on the belief in a just world scale were classified into four items measuring belief in ultimate justice and four items measuring belief in immanent justice, based on prior research (Umetani et al., 2020). Alpha coefficients were calculated for each subscale. The results showed sufficiently high reliability for belief in ultimate justice (*α* = 0.89) and belief in immanent justice (*α* = 0.74). Therefore, the four items for each subscale were summed to yield scores for belief in ultimate justice and belief in immanent justice.

### 3.2 Descriptive statistics for just-world beliefs, maternal non-textual utterances, and the number of days per week that the mothers read picture books

The variables analyzed included scores for belief in ultimate justice and belief in immanent justice, the total number of maternal extra-textual utterances during picture book reading, the number just-world belief-related utterances, their relative to total off-text maternal speech, and the number of days per week that the participating mothers read picture books. Mean values and standard deviations for these variables are presented in Table 1.

**Table 1:** Descriptive statistics of the variables used in this study.

Name of variable	Average	SD	Min	Max
Score on the scale of belief in ultimate justice	13.58	3.86	7.00	20.00
Score on the scale of belief in immanent justice	16.13	2.47	12.00	20.00
Total amount of maternal non-textual utterances	14.58	14.62	0.00	52.00
Number of utterances related to belief in a just world	3.12	4.13	0.00	19.00
The proportion of utterances related to belief in a just world within the total maternal non-textual utterances^1^	0.20	0.16	0.00	0.58
The number of days per week mothers read picture books	5.46	1.93	1.00	7.00

^1^Since the proportion could not be calculated for the three mothers who did not make any nob-textual utterances, it was calculated based on data from the remaining 21 mothers.

### 3.3 Associations between mothers’ just-world beliefs and non-text utterances during picture book Reading

To examine associations between maternal narrative additions and just-world belief scores, Spearman’s rank correlation coefficients were calculated with infant age as a control variable (Table 2). The total number of maternal non-textual utterances was significantly correlated with belief in immanent justice. By contrast, the number and proportion of just-world belief-related utterances showed significant positive correlations with belief in ultimate justice but not with belief in immanent justice.

**Table 2:** Spearman’s correlation coefficient between maternal non-textual utterances and scores on the scale of belief in a just world.

	Score on the scale of belief	
	Ultimate justice	Immanent justice
Total amount of maternal non-textual utterances	0.26	0.44*
Number of utterances related to belief in a just world	0.42*	0.36
Proportion of utterances related to belief in a just world	0.48*	0.25

*p < 0.05.

### 3.4 Associations between infant age and maternal just-world belief-related utterances

To examine whether infant age was associated with the proportion of just-world belief-related maternal utterances, we calculated Spearman’s rank correlation coefficient between these two variables. The results showed no significant correlation [*ρ*(20) = 0.27, n.s.].

### 3.5 Associations between the number of days per week mothers read picture books at home and maternal just-world belief-related utterances

To examine whether the number of the days per week mothers read picture books at home and the proportion of just-world belief-related maternal utterances, we calculated Spearman’s rank correlation coefficient between these two variables, with the infants’ age controlled. The results showed no significant correlation [ρ(18) = 0.23, n.s.].

## 4 Discussion

### 4.1 Factors influencing mothers’ just-world belief utterances during picture book reading

The present study examined the association between caregivers’ belief in a just world and maternal off-text utterances during shared picture book reading. The results showed that both the frequency and proportion of relevant maternal utterances were significantly associated with belief in ultimate justice, but not with infant age. These findings suggest that individual differences in maternal belief strength, rather than developmental stage, are related to how mothers verbally elaborate on picture book content.

This finding extends previous research demonstrating that caregivers’ parenting attitudes are reflected in their reading behaviors (Saito and Uchida, 2013) by showing, for the first time, that just-world beliefs are also implicated in these interactions. In particular, mothers with stronger beliefs in ultimate justice may be more likely to produce utterances that emphasize the narrative additions related to belief in just world. The present findings raise the possibility that caregivers’ language during everyday interactions, such as picture book reading, may constitute one pathway through which early-emerging just-world beliefs are supported or reinforced during infancy and toddlerhood.

Why did such beliefs influence mothers’ commentary during picture book reading with infants and toddlers? One possibility is that mothers with stronger beliefs produced more commentaries overall. Indeed, the degree of mothers’ belief in immanent justice was positively correlated with the total amount of off-script maternal speech. However, as described above, mothers’ belief in ultimate justice was specifically associated with both the number and proportion of maternal elaborations. This pattern suggests selective emphasis of just-world themes during narration.

Shimizu et al. (2018) showed that mothers made moral evaluations for their infants while watching videos depicting puppets helping or hindering others. Although picture books and videos differ in format, the present findings are consistent with Shimizu et al. (2018) in showing that caregivers provide moral evaluations of characters through media. Our study extends this work by demonstrating that caregivers with stronger just-world beliefs are more likely to produce additions to the narrative that morally evaluate characters and emphasize both positive and negative aspects of characters’ actions.

### 4.2 Associations between maternal non-textual utterances and belief in ultimate and immanent justice

Belief in a just world refers to the conviction that the world is a fair and safe place, where sudden misfortune does not occur and people receive what they deserve (Lerner, 1980). This belief is commonly divided into belief in ultimate justice, which holds that losses incurred due to misfortune will be compensated in the future, and belief in immanent justice, which holds that events result from past actions (Maes, 1998). This study found that the number of mothers’ extra-textual utterances related to such beliefs were significantly positively correlated with the strength of belief in ultimate justice, whereas no significant correlation was observed with belief in immanent justice. This suggests that mothers with stronger belief in ultimate justice are more likely to produce just-world belief-related utterances beyond the written text when reading picture books to infants and toddlers. Some mothers, despite making utterances beyond the written text, did not make any utterances related to the belief in a just world. Mothers who did not hold strong ultimate beliefs in a just world may have focused on finishing the picture book smoothly—such as by encouraging their children to read along—rather than emphasizing the depictions of a just world in the book.

Why was this association observed for belief in ultimate justice but not for belief in immanent justice? One explanation may relate to the narrative structure of picture books. The picture book *Anpanman* used in this study unfolds chronologically, encouraging readers to anticipate future developments rather than revisit earlier events. Belief in ultimate justice centers on expectations about future outcomes, and individuals with a stronger such belief may therefore be more inclined to predict positive resolutions before the story concludes. Prior research has shown that when individuals engage with narratives, they anticipate future developments based on their just-world beliefs. Callan et al. (2013), for example, measured participants’ gaze using eye tracking while they viewed images of a man posing victoriously or an overturned car while listening to stories describing the man as either kind to his wife or abusive toward her.

Analysis revealed that before hearing the story’s conclusion, participants who heard a story about a man who treated his wife kindly directed their gaze toward an image of the man making a victory pose, whereas participants who heard a story about a man who behaved violently toward his wife directed their gaze toward an image of an overturned car. This finding suggests that people expect positive outcomes for individuals who behave well and negative outcomes for those who behave poorly. Accordingly, mothers with strong belief in ultimate justice may emphasize characters’ moral or immoral behavior on the current page to encourage children to anticipate future outcomes depicted on subsequent pages.

The story of *Anpanman* is widely known in Japan and familiar to both mothers and children. Mothers therefore anticipate the conclusion in which “the hero Anpanman defeats the villain Baikinman.” Mothers with stronger belief in ultimate justice, who accept this resolution as appropriate, may speak negatively about the villain Baikinman, express concern for Anpanman during periods of misfortune, and emphasize injustice in ways that foreground his eventual triumph. In this study, mothers appeared to read aloud while anticipating outcomes in which “good things happen to characters who behave well, and bad things happen to characters who behave badly.” This expectation aligns with belief in ultimate justice, which holds that losses caused by injustice will be compensated in the future. Mothers who strongly endorsed this belief may therefore have emphasized scenes reflecting this worldview, such as “The mean Baikinman will get his comeuppance in the end” or “Anpanman, who was bullied by Baikinman, will be rewarded in the end.” To help children function smoothly as members of society, parents teach them the social customs shared within that society through their daily interactions (Callanan and Siegel, 2014). Mothers who strongly hold the belief in an ultimately just world are likely to view behavior consistent with that belief as socially desirable. For such mothers, stories depicting a worldview consistent with that belief may have served as suitable textbooks for teaching their children socially desirable behavior.

Research by Kanakogi et al. (2025) indicates that belief in immanent justice typically emerges around the latter half of age seven, whereas belief in ultimate justice is already observed by age five. They attribute this developmental difference to the maturation of temporal processing abilities. While young children can understand forward temporal relations from the present to the future around age four, they require more advanced development to process reverse temporal relations from the present to the past (Friedman, 2008). Consequently, belief in immanent justice, which requires past-oriented reasoning, may be more difficult for younger children to grasp, whereas belief in ultimate justice, which relies on future-oriented reasoning, may be accessible earlier. Kanakogi et al. (2025) argued that immaturity in temporal processing explains why belief in ultimate justice develops earlier than belief in immanent justice. By contrast, this study suggests that, in addition to temporal processing constraints, caregivers who strongly endorse belief in ultimate justice may actively emphasize and convey this worldview when young children encounter narratives structured around it. However, since this study did not examine the causal relationship between the strength of a mother’s belief in a just world and the development of her child’s belief in a just world, caution is warranted in interpreting these findings.

### 4.3 Study limitations and future research directions

This study is the first to demonstrate that individual differences in caregivers’ beliefs in a just world are reflected in how they emphasize characters or scenes during moralistic picture book reading with infants and toddlers. Given evidence that caregivers’ social evaluations of characters in videos are associated with infants’ acquisition of social evaluation (Shimizu et al., 2018), it is plausible that caregivers’ just-world beliefs are transmitted through their utterances during picture book and video viewing. However, this study did not directly examine whether such elaborations influence the development of infants’ and toddlers’ own just-world beliefs. In addition, this study focused on a single picture book with a moral theme, and the children who were read the book spanned a wide range of ages. Narratives related to belief in a just world are not limited to stories that reward good and punish evil but also include themes such as effort being rewarded or kindness returning to the self. Future research should examine whether caregivers’ reading styles vary across different types of just-world narratives. Longitudinal studies using diverse storybooks will be necessary to clarify how belief in a just world develops during infancy and toddlerhood. Moreover, caregivers will likely adjust their reading style to match the child’s language development and change the types of picture books they choose to match the child’s social development. While this study statistically controlled for the influence of the child’s age in its analysis, to obtain more accurate results, the age of the preschoolers should be standardized at the start of the observation period. Furthermore, as mentioned in the Procedure section, we did not impose any specific restrictions on how the participating mothers read the picture books. This was done to allow the mothers to adjust their reading style as they saw fit. However, given that this study took place in a setting where other mothers and children were present and might have been observing them, it cannot be ruled out that the participating mothers did not adopt their usual, natural reading style as they would at home, but instead adopted an unnatural and formal style. In addition, while all of the caregivers in this study were mothers, the primary reader of picture books at home is not necessarily the mother; it could also be the father or another adult. To examine whether daily caregiver -child picture book reading at home influences the development of children’s beliefs in a just world, it will be necessary to conduct observations of reading sessions occurring within the home setting in future studies.

Another important limitation of this study was that this study focused only on the Japanese parents. The fact that there are cultural differences in the degree to which people hold a just-world belief (Murayama et al., 2022; Murakami et al., 2022) suggests that each culture may have its own set of morally acceptable behaviors. Therefore, the emphasis on a worldview consistent with a just-world belief observed in this study among mothers may be unique to Japan. In the future, it will be necessary to observe picture book reading sessions between parents and children from other cultures and compare the results with those from Japan.

This study demonstrated that individual differences in caregivers’ belief in a just world influence the extent to which they produce belief-related utterances during picture book reading with moral themes of rewarding good and punishing evil. Picture book reading may therefore provide a context in which infants and toddlers begin to acquire caregivers’ just-world beliefs. This study could serve as an important first step toward examining the mechanisms by which parents’ just-world beliefs are passed on to their children through activities such as reading picture books together. The degree to which caregivers emphasize picture book depictions consistent with just-world beliefs varies depending on the strength of their own just-world beliefs. These differences may help explain the developmental origins of individual differences in just-world beliefs as a form of social cognition, particularly through interactions with infants’ emerging capacity for joint attention, an early-developing mechanism that appears around 9 months of age (Tomasello, 1999). Future longitudinal research should examine whether the frequency of caregivers’ just-world belief-related commentaries predicts the subsequent development of children’s own just-world beliefs.

## Data Availability

The raw data supporting the conclusions of this article will be made available by the authors, without undue reservation.
